# Cluster-tic syndrome as the initial manifestation of multiple sclerosis

**DOI:** 10.1007/s10194-012-0449-2

**Published:** 2012-04-28

**Authors:** V. González-Quintanilla, A. Oterino, M. Toriello, C. de Pablos, Y. Wu, E. de Marco, J. Pascual

**Affiliations:** University Hospital “Marqués de Valdecilla”, Santander, Cantabria Spain

## Abstract

We report the case of a patient diagnosed as having cluster-tic syndrome as the initial manifestation of multiple sclerosis (MS). The patient’s headache bouts improved after treatment with antiepileptic drugs, steroids, and beta-interferon. Magnetic resonance imaging (MRI) scans showed a pontine demyelinating lesion involving the area of the trigeminal root inlet and main sensory nucleus. Neurophysiological studies correlated well with MRI lesions. The association between cluster-tic syndrome and MS is an exception, and the mechanism of the pain is still unknown; therefore, this case might suggest a pathophysiological relationship between the trigeminal main sensory nucleus and cluster-tic syndrome.

## Introduction

Cluster-tic syndrome is characterized by the coexistence of three types of pain attacks. Pain resembling trigeminal neuralgia may be the first to occur or happen within or after the throbbing, cluster-type pain; the second, a cluster headache bout, and the third, mixed. These three pain types could be provoked by a trigger manoeuver. Although there is no proof for a common pathophysiology, both pain types always occur in the same facial side, often involving the same trigeminal territory, and could be elicited by the same manoeuver [1, 2]. A single lesion affecting the sensory trigeminal pathway is most often suspected, but rarely observed. Multiple sclerosis patients have a 20-fold increased risk for developing trigeminal neuralgia [3, 4]. The association with cluster headache is an exception, with only a few cases reported in the literature [5–7]. Gentile et al. [7] suggested that cluster headache may be generated primarily from within the brain, an idea concurring with the study by Matharu and Goadsby [8], showing the persistence of cluster headache attacks after complete surgical section of the trigeminal sensory roots.

This report describes the case of a 35-year-old male suffering from a cluster-tic headache as the initial manifestation of multiple sclerosis (MS). Neurophysiological and magnetic resonance imaging (MRI) findings in this case might illustrate the pathophysiology of cluster-tic headache.

## Case report

This 35-year-old male was admitted 6 years ago to the neurology outpatient clinic of our hospital suffering from left periocular neuralgiform pain for the last 2 months, consisting of paroxysmal attacks of stroke-like sensations, which developed spontaneously or were triggered by innocuous stimuli in facial and intraoral areas. Several seconds after that, he felt a throbbing retro-orbital pain with unilateral autonomic symptoms such as homolateral conjunctival injection, lacrimation, nasal congestion, rhinorrhoea, and palpebral fissure narrowing. This latter pain lasted less than 3 h, most often 30–40 min, and was successfully treated with subcutaneous sumatriptan injections (6 mg each) and oxygen at a rate of 10 l/min at the emergency room. With the cessation of cluster-type pain, the patient had a pain-free period which ranged from hours, at the beginning of the disease, to days or weeks, after treatment initiation. These bouts (neuralgiform and cluster types) were frequent (2–4/day) at regular hours (12:00 to 16:00 h in the daytime; 03:00 to 04:00 at night) before the use of carbamazepine. Carbamazepine, initially scheduled at 600 mg bid, was ineffective, as were non-steroidal anti-inflammatory drugs. Therefore, pregabaline (600 mg bid), clonazepam (6 mg tid), and gabapentine (800 mg bid) were tried in combination and with verapamil (360 mg bid). Some, but unsatisfactory, clinical benefit was achieved with verapamil (360 mg bid), pregabaline (600 mg bid), and carbamazepine (1,400 mg bid). These three drugs combined achieved the best clinical results (one or two cluster-tic bouts every 2 weeks). Nevertheless, three lidocaine endovenous boluses (500 mg/day for five consecutive days) were administered the following year, and thermocoagulation of his left Gasser ganglion was applied three times in the same period. Pain bouts disappeared for weeks after lidocaine boluses, but they relapsed with the same violence. Each of the thermocoagulation procedures was followed by a variable attack-free period, but polytherapy was still needed since attempts at dose-lowering proved useless. Neuralgiform attacks had always been sharp, stabbing pain in the periorbital region, lasting for seconds and always followed by cluster-type pain. After the first thermocoagulation of his left Gasser ganglion, cluster-type pain mostly disappeared. Then bouts of neuralgiform pain were not followed by cluster-type pain. There was no cluster-type pain recorded without the initial, neuralgiform-type pain; bouts always occurred on his left side. Most recently, some months after the third thermocoagulation procedure, neuralgiform bouts are less frequent (1–2 per month, lasting a few seconds). Even attempts at treatment simplification have not been useful. Repeated neurological exams were normal for the first 3 years, but generalized hyperreflexia, consisting of generalized brisk deep tendon reflexes and exhaustible clonus on his Achilles tendon, was progressively observed over the last 2 years. At present, other neurological symptoms and signs are absent, but fatigue has been a complaint recorded since beta-interferon was initiated. Cerebrospinal fluid (CSF) routinely performed 2 years after the initial symptoms (a spinal tap was proposed after the first MRI, but patient refused it) was normal, the oligoclonal bands being absent.

### Neuroimage studies

**Fig. 1:**
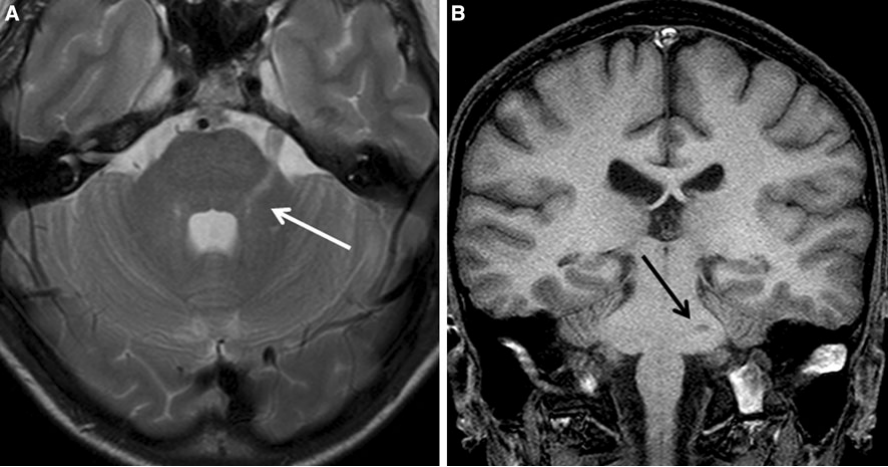
This figure represents the first MRI (without gadolinium) study performed on the patient: axial T2 (**a**) and coronal T1-weighted images (**b**) showing T2 hyperintense signals located at the territory of the* left* trigeminal root inlet and main sensory nucleus in the brainstem, which is hypointense in T1-weighted image coronal section (**b**)

**Fig. 2:**
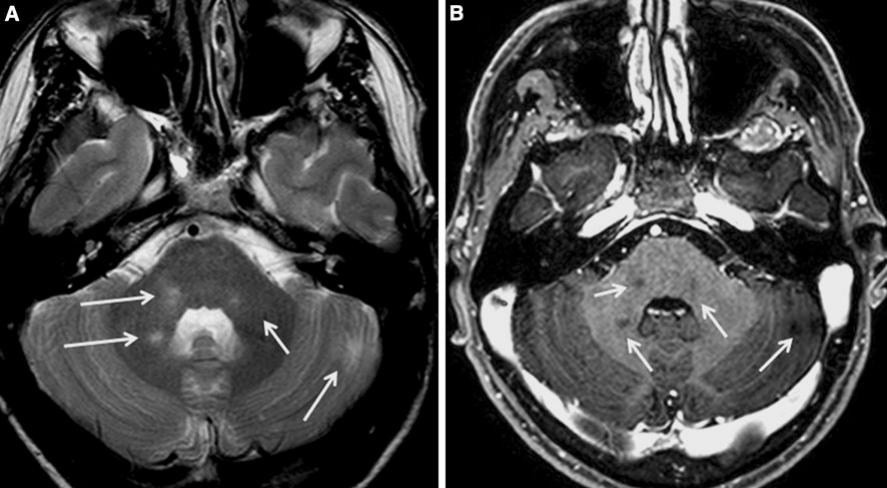
This MRI study was performed 2 years after that of Fig. 1 and showed new T2-weighted lesions, which are hypointense in T1 gadolinium-enhanced images in the pons and cerebellum (*arrows*). Hyperintense T2 lesions give a mirror image of the trigeminal root inlet and main sensory nucleus. **a** T2-weighted images; **b** T1 gadolinium-enhanced image

**Fig. 3:**
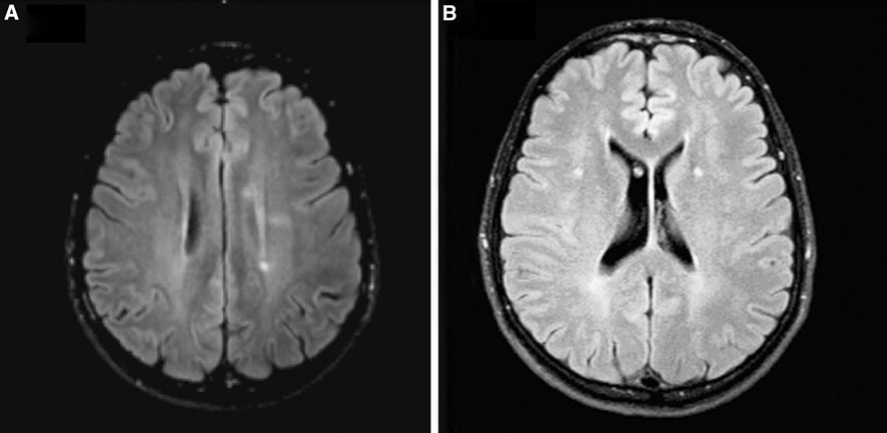
These supratentorial MRI images correspond to the same study as Fig. 2. Both T2-weighted image (**a**) and FLAIR (**b**) discovered new periventricular lesions

Computed tomography (CT) scan was normal. The first MRI study, performed 2 months after the initial symptoms, showed a T2-weighted hyperintense lesion (Fig. 1a) in the left trigeminal main sensory nucleus and root inlet, being hypointense (black hole) on T1-weighted image (Fig. 1b). In a second MRI study, performed 2 years later, eight T2-weighted hyperintense lesions were observed, some of them in the left trigeminal root inlet and main sensory nucleus area (Fig. 2a), but also in the right side, giving a mirror image of trigeminal root inlets and main sensory nucleus, in the left median pedunculus, and in the left cerebellar hemisphere. All of these lesions were hypointense in gadolinium-enhanced T1-weighted images (Fig. 2b). In the same MRI study, we observed new MRI lesions (Fig. 3a, T2-weighted image; Fig. 3b, FLAIR). 6 months later, a further MRI study showed new, periventricular, and juxtacortical lesions (data not shown). Based on the MRIs a diagnosis of MS was considered, and the patient was treated accordingly.

### Neurophysiological studies

**Fig. 4:**
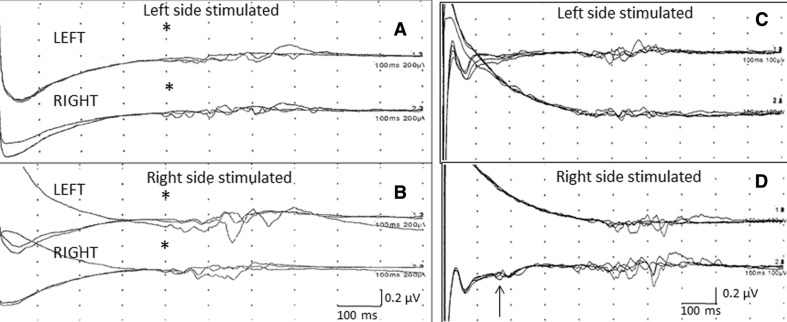
This figure represents the first blink-reflex performed on the patient. After stimulation with superficial electrodes, both R1 components were absent (**a**, *left*; **b**, *right*), while both R2 components were normal (*asterisks*; latencies ranged from 35.4 to 41.0 ms). The second study (**c**, **d**) was performed 5 years after the first. There was no variation in shape or latencies of R2 components obtained after stimulation of both supra-orbital nerves, but a small and delayed R1 component is visible after stimulation on the* right* supra-orbital nerve (**d**, *arrow*)

The first blink-reflex study, performed 2 months after the initial symptoms, showed an absence of R1 component after stimulation of both supra-orbital nerves, the R2 component being bilaterally normal (Fig. 4a, b, asterisks. Latencies ranged from 35.4 to 41.0 ms). Trigeminal sensory-evoked potentials showed a delay of cortical latencies after left mandibular nerve stimulation (P20 wave at 38.45 ms; data not shown). Five years later, a new and recent blink-reflex study showed the R1 component absent after left supra-orbital nerve stimulation (Fig. 4c), but a small and delayed R1 component could be seen after right supra-orbital nerve stimulation (Fig. 4d, arrow). After treatment with methylprednisolone (MTP 1 g/day for 5 days) pain bouts improved, and their regular pattern changed to random along the day. MTP was used five times throughout his illness. Some weeks after the beginning of BIF-1A (250 mcg/three times a week) injections, pain bouts temporarily disappeared. Carbamazepine was reduced to 600 mg bid and maintained today. In addition, there were normal parameters in the somatosensory evoked potentials studied at both median nerves and both lower limbs.

## Discussion

We report here the case of a patient suffering from cluster-tic syndrome. This rare headache variant was the initial presenting symptom of MS. The patient was diagnosed as having MS according to the new McDonald’s criteria (2011 revision)[9], although we used Barkoff–Tintoré criteria before, which were the most specific MRI criteria available for assessing the risk of conversion to MS after clinically isolated syndromes [10, 11]. This patient had had at least one objective—clinically and neurophysiologically demonstrated—lesion, two abnormal signs (pyramidalism; trigeminal lesion), and at least two new T2 lesions on MRI scans; therefore, the McDonald criteria for category III was achieved. The initial trigeminal lesion might generate the series of events which are thought to give rise to cluster-tic syndrome through trigeminal-vascular system activation. Involved structures correlated well with the absence of the left blink-reflex R1 component. The early reflex (R1) is mediated via the main sensory nucleus, while the late responses are probably transmitted via multi-synaptic pathways which include, at least, the spinal tract and nucleus [12, 13]. Hence the absence of R1 suggests an ipsilateral pontine lesion, provided that facial and trigeminal nerves remain intact [14, 15]. The absence of the right R1, in the first study, is astonishing. We performed the blink-reflex test for the first time when the first MRI study was done. In this first MRI, no demyelinating lesions were noted in the right pons, but 2 years later, the same lesion appeared in the right trigeminal sensory nucleus and root inlet as had previously been observed in the left. Therefore, we might speculate that unrevealed lesions, strategically located at the opposite side, could explain the right R1 absence as a subclinical lesion. This hypothesis is supported by the partial recovery experienced after the beginning of treatment with BIF-1A. Bilaterally normal R2 components reflect the integrity of the trigeminal spinal tract and the corresponding fiber root inlets. The main trigeminal sensory nucleus has an important role in connecting trigeminal inputs with the opposite trigeminal nucleus, facial nucleus, locus coeruleus, and supratentorial nuclei, including thalamus and hypothalamus. Lesions affecting trigeminal root fibers directed to the main sensory nucleus will affect the blink-reflex R1 component, leaving the R2 component unaltered and this fact could contribute to localize the lesion. It is possible that trigeminal deafferentization of parasympathetic nuclei and hypothalamus might contribute to the cluster headache through trigeminal-vascular reflex activation in this patient. Our observations implicate a central origin for the pain in this patient, supporting the hypothesis that cluster-tic syndrome is generated primarily in the brain. With data presented here, we cannot address as to why having both trigeminal nucleus and root inlets affected in MRI scans (Fig. 2), this patient had pain only in his left trigeminal area.

Finally, after treatment with antiepileptic drugs, steroids and beta-interferon, the patient improved, but still needs polytherapy for pain control. We emphasize here the importance of serial MRI scans in young people suffering from trigeminal neuralgia to assess the final diagnosis of MS.

## References

[1] Watson P, Evans R (1985). Cluster-tic syndrome. Headache.

[2] Alberca R, Ochoa JJ (1994). Cluster-tic syndrome. Neurology.

[3] Katusic S, Williams DB, Beard CM, Bergstraldh EJ, Kurland LT (1991). Epidemiology and clinical features of idiopathic trigeminal neuralgia and glossopharyngeal neuralgia: similarities and difference. Rochester Minnesota. 1945–1984. Neuroepidemiology.

[4] Cruccu G, Biasiotta A (2009). Trigeminal neuralgia and pain related to multiple sclerosis. Pain.

[5] Leandri M, Cruccu G, Gottlieb A (1999). Cluster headache like pain in multiple sclerosis. Cephalalgia.

[6] Then Berg F, Dose T, Forderreuther S (2000). Symptomatischer clusterkopfschmerz. Ausdruck eines MS-Schubes mit kernspintomographischem N achweis einer pontomedulären Läsion des ipsilateralen trigeminuskerngebietes. Nervenarzt.

[7] Gentile S, Ferrero M (2007). Cluster headache attacks and multiple sclerosis. J Headache Pain.

[8] Matharu MS, Goadsby PJ (2002). Persistence of attacks of cluster headache after trigeminal nerve root section. Brain.

[9] Polman CH, Reingold SC, Bandwell B (2011). Diagnostic criteria for multiple sclerosis: 2010 revisions to the McDonald criteria. Ann Neurol.

[10] Barkhof F, Filippi M (1997). Comparison of MRI criteria at first presentation to predict conversion to clinically definite multiple sclerosis. Brain.

[11] Sastre-Garriga J, Tintoré M (2004). Specifity of Barkhof criteria in predicting conversion to multiple sclerosis when applied to clinically isolated brainstem syndromes. Arch Neurol.

[12] Tokunaga A, Oka M, Murao T (1958). A experimental study on facial reflex by evoked electromyography. Mej J Osaka Univ.

[13] Kennelly KD (2006). Electrophysiological evaluation of cranial neuropathies. Neurologist.

[14] Kimura J (1970). Alteration of the orbicularis oculi reflex by pontine lesions; study in multiple sclerosis. Arch Neurol.

[15] Kimura J (1975). Electrically elicited blink reflex in diagnosis of multiple sclerosis. Brain.

